# Coordination Decisions for a Low-Carbon Supply Chain Considering Risk Aversion under Carbon Quota Policy

**DOI:** 10.3390/ijerph19052656

**Published:** 2022-02-24

**Authors:** Hao Zou, Jin Qin, Xiaofeng Long

**Affiliations:** 1 School of Traffic and Transportation Engineering, Central South University, Changsha 410075, China; zouhaocm@csu.edu.cn; 2 School of Business Administration, Hunan University of Finance and Economics, Changsha 410205, China; longxiaofeng@gmail.com

**Keywords:** low-carbon supply chain, risk aversion, carbon quota, cost-sharing contract

## Abstract

To mitigate climate change, the governments of various countries have formulated and implemented corresponding low-carbon emission reduction policies. Meanwhile, consumers’ awareness of the necessity of environmental protection is gradually improving, and more consumers pay attention to the environmental attributes of products, which all encourages enterprises to have great power to implement low carbon technology. As rational decision makers, members tend to show the characteristics of risk aversion. How to meet the needs of consumers and reduce their own risks has become a key point of low-carbon supply chain management. Considering carbon quota policy, in this paper, the optimal pricing decision-making process of a supply chain system is discussed under risk-neutral and risk-avoidance decision-making scenarios by game theory, and a cost-sharing contract is used to coordinate the decision-making process of a supply chain system. By analyzing the influence of the risk aversion coefficient on the optimal strategies of participants, we find that when the manufacturer has the risk aversion characteristic, the risk aversion coefficient will further reduce the carbon emission rate, the wholesale price of the product and the manufacturer’s profit but increase the product order quantity and the retailer’s profit. In addition, if consumers have a high preference for low-carbon products, the manufacturer’s risk-aversion coefficient will lead to a lower selling price than in the centralized decision-making situation, and the profit of the supply chain system will also be further reduced. When the cost-sharing contract is adopted for coordination, the Pareto improvement of supply chain members’ profits can be achieved when the parameters of the cost-sharing contract are appropriate, regardless of the manufacturer’s risk-neutral decision or risk-aversion decision.

## 1. Introduction

Existing studies have shown that global warming is directly related to carbon and other greenhouse gas emissions [1]. It poses a huge threat to the survival and health of human beings and has attracted wide attention from all countries in the world [2]. Many countries try to control carbon emissions through legislation. For example, the Kyoto Protocol in 1997 [3], the Copenhagen Accord in 2009 [4] and the Paris Agreement in 2015 [5]. However, the total global greenhouse gas emissions have not been reduced [6]. According to the data released by the Global Carbon Project, the total carbon dioxide emissions from global energy consumption increased by 1.7% in 2018 [7]. How to effectively reduce carbon emissions will be the focus of global attention. To mitigate climate change, governments of various countries have formulated and implemented corresponding low-carbon emission reduction policies, such as carbon taxes and cap-and-trade systems [8,9]. Compared with command-and-control standards, carbon cap-and-trade mechanisms are more effective in reducing carbon emissions [10]. For example, the EU Emissions Trading Scheme [11], China’s cap-and-trade system [12], and South Korea’s carbon trading system [13] are all successful solutions.

Due to uncertain demand and other factors, manufacturing enterprises tend to show the characteristics of risk aversion in their supply chain management [14]. At this point, the assumption of risk neutrality is insufficient to apply to contemporary supply chain management [15]. For example, JC Penney in the US adopts a conservative ordering strategy due to financial pressure [16]. Coca-Cola Company takes into account consumers’ preference for low-sugar products and adopts a risk-reduction investment strategy [17]. Generally, the utility of supply chain members is an increase function of expected profit, but it decreases with the risk-sensitive function [18]. Therefore, it is particularly important to balance expected profits and risk aversion [19]. In summary, the greenhouse effect caused by carbon emissions has become increasingly prominent, and many countries have developed and implemented cap-and-trade systems. Meanwhile, consumer awareness of environmental protection is enhanced, and as more consumers pay attention to the environmental attributes of products. In real life, the policy makers tend to avoid risk to reduce losses. However, there is little literature on the impact of carbon quotas and risk aversion on the optimal price and coordination strategies of participants in a low-carbon supply chain. Based on this, we investigate the effects of risk aversion on equilibrium decision making and participant profit and implement a system Pareto improvement through a cost-sharing contract. Our research attempts to solve the following questions: (1) How does risk aversion affect the pricing and profit of a low-carbon supply chain under carbon quota regulations? (2) What is the impact of consumers’ preference for low-carbon products and manufacturers’ risk aversion on supply chain decision-making? (3) How can contract parameters be designed to achieve Pareto improvement of members’ profits in a low-carbon supply chain?

Consumers’ low-carbon preference and carbon quota policy give manufacturers the motivation to reduce emissions. At the same time, manufacturers show risk aversion due to the additional costs of emission reduction investments. At this point, how manufacturing enterprises make decisions to meet the government’s emission requirements and obtain certain product market demand is of great significance. Compared with the existing literature, this paper combines carbon quota policy and consumers’ low-carbon preference into the decision-making process of low-carbon supply chain and explores the decision-making problem of manufacturers’ risk-averse behavior due to the investment in emission reduction technologies. We use game theory to discuss the optimal pricing decision-making process of supply chain systems under risk-neutral and risk-averse decision-making scenarios and use a cost-sharing contract to coordinate the decision-making process of supply chain system, and obtain the specific value range of a cost-sharing coefficient and related management enlightenment.

The structure of the rest of the paper is as follows. Section 2 conducts the comprehensive literature review on the carbon quota, risk avoidance and contract coordination based on the low-carbon supply chain. Section 3 builds pricing decision models under different circumstances and solves them. Section 4 is numerically analyzed, and the results are discussed. Meanwhile, a sensitivity analysis of the relevant parameters is carried out. Section 5 summarizes the research in this paper and proposes future research directions.

## 2. Literature Review

The previous studies related to our study can be classified to three categories, which is reviewed in a way that usually appears in literature reviews [20,21,22]. One is the study of supply chain decision-making under carbon policy. The second is the study of low carbon supply chain decision-making considering risk aversion. The third category is the research on coordination decision of low carbon supply chain contract. This paper focuses on reviewing the relevant literature.

### 2.1. Research on Supply Chain Decision-Making under Carbon Policy

In order to effectively reduce the carbon emissions of enterprises, the government has implemented carbon taxes [9], cap and trade system [1,12]. How to make investment decisions on emission reduction technologies based on carbon policies is an important part of current enterprise operation management. At present, the research of supply chain decisions based on carbon tax mainly includes enterprise pricing [23,24], contract coordination [25], and dual channels [26]. In addition, some researchers have compared and analyzed the decision-making process of supply chain under carbon tax and cap-and-trade system. Xu et al. [27] studied the joint pricing of manufacturing enterprises under two types of regulation and analyzed the impact of carbon tax and total cap on corporate profits and social welfare. Hu et al. [5] further extended the traditional supply chain to the closed loop system and analyzed the impact of carbon tax and carbon quota policy on enterprise profits, and the results show that carbon quota policy is more suitable for remanufacturing.

Compared with carbon tax regulation, the cap-and-trade system is an effective mechanism to achieve emission reduction targets. In recent years, many researchers have studied it from different angles. In terms of production decision-making, Zhang et al. [1] studied the production planning of multiple products with random demand and analyzed the impact of carbon cap on production decision-making and corporate profits. Later, Du et al. [28] further considered the impact of carbon footprint and low-carbon preference and constructed the optimal production decision model. In terms of ordering decision, He et al. [29] studied the optimal ordering problem under total quantity control based on the economic ordering batch model. Ji et al. [30] further extended to the O2O retail supply chain and studied the influence of carbon trading cap on the emission reduction decision and optimal ordering of supply chain members. In terms of coordination decision-making, Bai et al. [31] designed the coordination contract based on income and investment sharing through the comparative analysis of profit and carbon emissions. Xu et al. [32] further considered the influence of low-carbon preference and channel substitution and constructed the dual-channel emission reduction decision model by using game theory. There are literatures on supply chain decision-making based on carbon policy, which seldom consider the influence of risk aversion and other behavioral factors. In contrast, this paper focuses on the influence of manufacturer’s risk-avoidance behavior on decision making and the reasonable setting of contract parameters.

### 2.2. Research on Low-Carbon Supply Chain Decision-Making Considering Risk Aversion

Simon [33] put forward the theory of bounded rationality considering behavioral factors such as psychology and cognition of decision-makers. Later, Gino and Pisano [34] introduced the behavior and cognitive factors of decision-makers into the process of enterprise operation management, promoting the development of the field of behavioral operation management. Behavioral theory analysis points out that when decision-makers pay attention to their own benefits, they will also choose how to avoid their own risks. Due to the uncertainty of demand and other factors, enterprises often show risk- aversion characteristics in their supply chain management. Currently, mean-variance method [35], value-at-risk method [36,37] and conditional value-at-risk method [15,38] are the main risk avoidance measures of supply chain. Many scholars have introduced risk-avoidance behavior into the decision-making process of supply chain management and explored the impact of risk avoidance characteristics on pricing and coordination decisions of supply chain members [16,39,40,41]. In fact, due to the introduction of carbon policy, low-carbon technology investment and low-carbon preference, low-carbon supply chain is more complex than a traditional supply chain, and members pay more attention to risk avoidance when making decisions. At present, most existing literatures use the mean-variance method to measure the risk preference of low-carbon supply chain members. Wang et al. [42] analyzed the impact of decision makers’ risk avoidance characteristics on the performance of low-carbon supply chain system by comparing the decision-making process under the risk-neutral and risk-avoidance scenarios. Further, Bai et al. [14] and Sun et al. [43] studied the influence of a risk-aversion coefficient on carbon emission reduction rate and price considering horizontal competition among manufacturers. However, they did not focus on supply chain coordination. Later, Xing et al. [44] introduced risk avoidance into the closed-loop supply chain system and studied the influence of carbon emission trading price on supply chain and member performance. In contrast, we focus on manufacturer risk aversion and system coordination processes. In addition, a few scholars have studied the decision-making problem of low-carbon supply chain based on conditional value at risk. Deng et al. [45] constructed three emission reduction modes using Stackelberg game model and used CVaR criterion to measure the risk-aversion characteristics of retailers. Qi et al. [46] introduced cap-and-trade system into low-carbon supply chain and studied the impact of risk aversion on supply chain financial decision-making. In general, there is little literature on introducing risk aversion into low-carbon supply chain decision-making processes. Based on carbon quota policy and manufacturer’s risk avoidance behavior, this paper focuses on manufacturer’s emission reduction decision and system profit distribution.

### 2.3. Research on Contract Coordination of Low-Carbon Supply Chain

As independent individuals, supply chain enterprises making decisions from the perspective of a rational economic man will reduce their respective performance [47]. The supply chain contract guides all participants to make decisions through agreed contracts, which reduces the unnecessary costs of the system and ensures profit increase of all parties, thus becoming an effective way to realize supply chain coordination [48]. Supply chain management becomes more and more complicated due to the incorporation of carbon as an endogenous variable into the decision-making process of the supply chain. At this point, how to motivate supply chain enterprises to reduce emissions and coordinate the interests of all parties is of great significance. At present, some scholars have introduced quantity discount contract [49], revenue sharing contract [50], cost-sharing contract [51] into the decision-making process of low-carbon supply chain coordination. Existing studies on contract coordination of the low-carbon supply chain can be divided into three types: single contract coordination decision [3,52], improved single contract coordination decision making [32,53], multi-contract joint coordinated decision making [25,54]. However, they do not pay attention to the risk aversion of supply chain members. Wang et al. [42] and Deng et al. [45] studied the influence of risk avoidance coefficient of suppliers and manufacturers on the performance of low-carbon supply chain considering different carbon emission reduction subjects. Bai et al. [17] introduced carbon tax policy into low-carbon supply chain and studied the impact of risk avoidance on decision-making of supply chain system and realized system coordination through contract. On this basis, our research focuses on the impact of cap-and-trade system.

To sum up, some scholars have studied the pricing and coordination of low-carbon supply chains under the situation of risk aversion of enterprises in supply chain nodes, but few have included carbon quota policy and risk aversion in the decision-making process of low-carbon supply chains simultaneously. Qi et al. [46] introduces the cap-and-trade system into the decision-making process of a low-carbon supply chain by considering the risk aversion characteristics of manufacturers and capital-constrained retailers and study the impact of working capital, risk aversion degree and carbon trading price on the operation of the supply chain but do not pay attention to the coordination decision-making process of a low-carbon supply chain. Bai et al. [14] studied the impact of sustainable technology on improving the economic and environmental performance of the supply chain by comparing manufacturers with or without sustainable technology investment. In contrast, our study takes into account consumer preferences for low-carbon products and a cap-and-trade system and uses cost-sharing contracts to achieve Pareto improvements in supply chain members’ profits.

## 3. Model Construction and Result Analysis

### 3.1. Symbol Description and Model Assumptions

This paper considers a two-stage low carbon supply chain consisting of a manufacturer and a retailer. Among them, the manufacturing process of the manufacturer produces carbon emissions. In the beginning, the government gave the manufacturers a certain amount of carbon allowances free of charge. To keep within the quota, the manufacturers adopted advanced technologies to reduce their carbon emissions. If the manufacturer produces more carbon emissions than the government gives it, it will have to buy carbon emission rights from the carbon trading market. If manufacturers produce less carbon emissions than the government has given them, they can sell the excess credits. Manufacturers, as leaders in the Stackelberg game, set the wholesale price of products and the level of carbon emissions. Retailers act as followers to determine the selling price of products.

In order to clearly describe the constructed decision model, relevant parameters and decision variables are shown in Table 1.

Next, we make the following Hypothesis:

**Hypothesis** **1** **(H1).** *Market demand faced by retailers is not only related to sales prices but also affected by consumers’ preference for low carbon. Referring to Zhou et al. [55], the market demand function is described as q=s−bp+λβ.*

**Hypothesis** **2** **(H2).** *To reduce carbon emissions during production and processing, manufacturers need to introduce or improve technologies at a certain emission reduction cost. Referring to Swami et al. [56], the manufacturer’s carbon emission reduction cost is described as $C=k\beta^{2}/2$.*

### 3.2. Pricing Decision Models and Solutions under Different Circumstances

#### 3.2.1. Centralized Decision Model

In the decision-making process of centralized control, manufacturers and retailers as a whole face the sales market, and the supply chain can achieve vertical integration. At this point, the system determines the optimal product sales price p and the emission reduction rate of unit product β according to the market demand q, and its expected profit function can be expressed as:

(1) $$E(\pi_{sc}^{*})=(p-c)(\bar{s}-bp+\lambda \beta )+p_{c}[A-e(1-\beta )(\bar{s}-bp+\lambda \beta )]-\frac{1}{2}k\beta^{2}$$

We can get the following result by Formula (1).

**Proposition** **1.** *When $2bk-{\left(\lambda +p_{c}eb\right)}^{2}>0$, there exist unique p and β that maximize the expected profit of the supply chain system. At this point, the optimal product sales price $p^{*}$, emission reduction rate per unit product $\beta^{*}$, market demand $q^{*}$ and expected profit $E(\pi_{sc}^{*})$ of the system can be, respectively expressed as:*

(2) $$p^{*}=\frac{k(\bar{s}+bc+p_{c}eb)-(\lambda +p_{c}eb)(p_{c}e\bar{s}+p_{c}e\lambda +c\lambda )}{2bk-{\left(\lambda +p_{c}eb\right)}^{2}}$$

(3) $$\beta^{*}=\frac{\left(\bar{s}-bc-p_{c}eb)(\lambda +p_{c}eb\right)}{2bk-{\left(\lambda +p_{c}eb\right)}^{2}}$$

(4) $$q^{*}=\frac{bk(\bar{s}-bc-p_{c}eb)}{2bk-{\left(\lambda +p_{c}eb\right)}^{2}}$$

(5) $$E(\pi_{sc}^{*})=\frac{k{\left(\bar{s}-bc-p_{c}eb\right)}^{2}}{2[2bk-{\left(\lambda +p_{c}eb\right)}^{2}]}+p_{c}A$$

At the same time, to ensure that the variable is not negative, it must meet the following requirements: $k(\bar{s}+bc+p_{c}eb)-(\lambda +p_{c}eb)(p_{c}e\bar{s}+p_{c}e\lambda +c\lambda )>0$.

The proof of Proposition 1 is given in Appendix A.

Proposition 1 shows the existence and uniqueness of low-carbon supply chain system decisions under a carbon cap-and-trade policy. Obviously, the optimal pricing of the system is related to consumers’ low carbon preference and carbon trading price. In the following section, we establish the best operating decisions of members under risk neutrality and risk aversion and compare and analyze the relationship between parameters and profits.

#### 3.2.2. Decentralized Decision Model When the Manufacturer Is Risk Neutral

When making decentralized decisions, the manufacturer and retailer follow the Stackelberg game process. In other words, the manufacturer first determines w and β, and then the retailer determines p. At this point, the expected profit function of manufacturers and retailers can be, respectively expressed as:

(6) $$E(\pi_{m}^{n})=(w-c)(\bar{s}-bp+\lambda \beta )+p_{c}[A-e(1-\beta )(\bar{s}-bp+\lambda \beta )]-\frac{1}{2}k\beta^{2}$$

(7) $$E(\pi_{r}^{n})=(p-w)(\bar{s}-bp+\lambda \beta )$$

We can obtain the following result by Formulas (6) and (7).

**Proposition** **2.** *When $2bk-{\left(\lambda +p_{c}eb\right)}^{2}>0$, the manufacturer’s risk-neutral decentralized decision model has a unique product wholesale price w, emission reduction rate per unit product β and product sales price p so that the expected profit of manufacturers and retailers can reach the maximum. At this point, the optimal wholesale price of product $w^{n}$, emission reduction rate of unit product $\beta^{n}$, product sales price $p^{n}$ and market demand $q^{n}$ can be, respectively expressed as:*

(8) $$w^{n}=\frac{2k(\bar{s}+bc+p_{c}eb)-(\lambda +p_{c}eb)(p_{c}e\bar{s}+p_{c}e\lambda +c\lambda )}{4bk-{\left(\lambda +p_{c}eb\right)}^{2}}$$

(9) $$\beta^{n}=\frac{\left(\bar{s}-bc-p_{c}eb)(\lambda +p_{c}eb\right)}{4bk-{\left(\lambda +p_{c}eb\right)}^{2}}$$

(10) $$p^{n}=\frac{k(3\bar{s}+bc+p_{c}eb)-(\lambda +p_{c}eb)(p_{c}e\bar{s}+p_{c}e\lambda +c\lambda )}{4bk-{\left(\lambda +p_{c}eb\right)}^{2}}$$

(11) $$q^{n}=\frac{bk(\bar{s}-bc-p_{c}eb)}{4bk-{\left(\lambda +p_{c}eb\right)}^{2}}$$

At the same time, to ensure that the variable is not negative, it must meet the following requirements: $k(\bar{s}+bc+p_{c}eb)-(\lambda +p_{c}eb)(p_{c}e\bar{s}+p_{c}e\lambda +c\lambda )>0$, $\bar{s}-bc-p_{c}eb>0$.

The proof of Proposition 2 is given in Appendix B.

Proposition 2 shows the existence and uniqueness of supply chain members’ decisions when the manufacturer’s risk is neutral and decentralized. Retailers as followers determine the optimal wholesale price of products, and manufacturers make the optimal decision through the wholesale price and carbon emission reduction rate.

We input $w^{n}$, $\beta^{n}$ and $p^{n}$ into $E(\pi_{r}^{n})$, and $E(\pi_{r}^{n})$ can obtain the expected profit function of retailers and manufacturers under the manufacturer’s risk-neutral decentralized decision. Furthermore, the expected profit function of the whole system can be obtained as follows:

(12) $$E(\pi_{r}^{n})=\frac{bk^{2}{\left(\bar{s}-bc-p_{c}eb\right)}^{2}}{{\left[4bk-{\left(\lambda +p_{c}eb\right)}^{2}\right]}^{2}}$$

(13) $$E(\pi_{m}^{n})=\frac{k{\left(\bar{s}-bc-p_{c}eb\right)}^{2}}{2[4bk-{\left(\lambda +p_{c}eb\right)}^{2}]}+p_{c}A$$

(14) $$E(\pi_{sc}^{n})=E(\pi_{r}^{n})+E(\pi_{m}^{n})=\frac{k{\left(\bar{s}-bc-p_{c}eb\right)}^{2}(6bk-{\left(\lambda +p_{c}eb\right)}^{2})}{2{\left[4bk-{\left(\lambda +p_{c}eb\right)}^{2}\right]}^{2}}+p_{c}A$$

#### 3.2.3. Decentralized Decision Model in Manufacturer Risk Avoidance

Manufacturers have the characteristics of risk aversion, and retailers have the characteristics of risk neutrality. At this point, manufacturers will think not only about their own profits but also how to avoid risk. We use the mean variance method to measure the manufacturer’s expected utility [25]. By introducing the manufacturer’s risk-aversion coefficient η>0, the utility function of the manufacturer and the retailer can be expressed as:

(15) $$U(\pi_{m}^{m})=E(\pi_{m}^{m})-\eta \sqrt{VarE(\pi_{m}^{m})}=[w-c-p_{c}e(1-\beta )](\bar{s}-bp+\lambda \beta )+p_{c}A-\frac{1}{2}k\beta^{2}-\eta \delta_{s}[w-c-p_{c}e(1-\beta )]$$

(16) $$U(\pi_{r}^{m})=E(\pi_{r}^{m})=(p-w)(\bar{s}-bp+\lambda \beta )$$

We can obtain the following results by using Formulas (15) and (16).

**Proposition** **3.** *When $2bk-{\left(\lambda +p_{c}eb\right)}^{2}>0$, the manufacturer risk aversion decentralized decision model has a unique product wholesale price w, emission reduction rate per unit product β, and product sales price p to maximize the expected profit of manufacturers and retailers. At this point, the optimal wholesale price of product $w^{m}$, emission reduction rate of unit product $\beta^{m}$, product sales price $p^{m}$ and market demand $q^{m}$ can be, respectively expressed as:*

(17) $$w^{m}=\frac{2k(\bar{s}+bc+p_{c}eb-2\eta \delta_{s})-(\lambda +p_{c}eb)(p_{c}e\bar{s}+p_{c}e\lambda +c\lambda -2\eta \delta_{s}p_{c}e)}{4bk-{\left(\lambda +p_{c}eb\right)}^{2}}$$

(18) $$\beta^{m}=\frac{\left(\bar{s}-bc-p_{c}eb-2\eta \delta_{s})(\lambda +p_{c}eb\right)}{4bk-{\left(\lambda +p_{c}eb\right)}^{2}}$$

(19) $$p^{m}=\frac{bk(3\bar{s}+bc+p_{c}eb-2\eta \delta_{s})-(\lambda +p_{c}eb)(p_{c}eb\bar{s}+p_{c}eb\lambda +cb\lambda -p_{c}eb\eta \delta_{s}+\eta \delta_{s}\lambda )}{b[4bk-{\left(\lambda +p_{c}eb\right)}^{2}]}$$

(20) $$q^{m}=\frac{bk(\bar{s}-bc-p_{c}eb)+\eta \delta_{s}[2bk-{\left(\lambda +p_{c}eb\right)}^{2}]}{4bk-{\left(\lambda +p_{c}eb\right)}^{2}}$$

When $2k(\bar{s}+bc+p_{c}eb-2\eta \delta_{s})-(\lambda +p_{c}eb)(p_{c}e\bar{s}+p_{c}e\lambda +c\lambda -2\eta \delta_{s}p_{c}e)>0$ and $\bar{s}-bc-p_{c}eb>2\eta \delta_{s}$, the variable is nonnegative, so we can obtain $\eta <\left(\bar{s}-bc-p_{c}eb\right)/\left(2\delta_{s}\right)$.

The proof of Proposition 3 is given in Appendix C.

Proposition 3 indicates the existence and uniqueness of supply chain member decisions when the manufacturer makes decentralized decisions on risk aversion. Equations (17)–(19) show that the wholesale price of product $w^{m}$, the emission reduction rate of unit product $\beta^{m}$ and the sales price of product $p^{m}$ are all related to the manufacturer’s risk-aversion coefficient η. In the next section, we will verify the specific relationship between each parameter and η.

Let bk=B, ${\left(\lambda +p_{c}eb\right)}^{2}=C$, $(\bar{s}-bc-p_{c}eb)=S$ and $\eta \delta_{s}=H$, and input $w^{m}$, $\beta^{m}$ and $p^{m}$ into $U(\pi_{r}^{m})$ and $U(\pi_{m}^{m})$ to obtain the retailer and manufacturer’s utility function under the decentralized decision of the manufacturer’s risk avoidance and further obtain the overall system’s utility function as follows:

(21) $$U(\pi_{r}^{m})=\frac{{\left[BS+H(2B-C)\right]}^{2}}{b{\left(4B-C\right)}^{2}}$$

(22) $$U(\pi_{m}^{m})=\frac{k{\left(S-2H\right)}^{2}}{2[4B-C]}+p_{c}A$$

(23) $$U(\pi_{sc}^{m})=\frac{k(S-2H)[S(6B-C)+2H(2B-C)]}{2{\left(4B-C\right)}^{2}}+\frac{\eta^{2}\delta_{s}^{2}}{b}+p_{c}A$$

**Proposition** **4.** The wholesale price of the manufacturer is lower than that of the manufacturer in the risk-neutral diversification decision. The carbon emission reduction rate is smaller than that of risk-neutral decentralized decision making and smaller than that of centralized decision making. At the same time, the order quantity of the optimal product $q^{m}$ is larger than the order quantity $q^{n}$ under the risk-neutral decentralized decision but less than the order quantity $q^{*}$ under the centralized decision.

**Proof.**  By comparing $w^{m}$ and $w^{n}$, we can get: $w^{m}-w^{n}=\frac{-2\eta \delta_{s}[2k-(\lambda +p_{c}eb)p_{c}e]}{4bk-{\left(\lambda +p_{c}eb\right)}^{2}}$. According to the above analysis, the conditions for the feasibility and nonnegative existence of w and β are $2bk-{\left(\lambda +p_{c}eb\right)}^{2}>0$, so $w^{m}<w^{n}$ is easily obtained. Comparing $\beta^{*}$, $\beta^{n}$ and $\beta^{m}$, there are $\frac{\beta^{n}}{\beta^{*}}=\frac{2bk-{\left(\lambda +p_{c}eb\right)}^{2}}{4bk-{\left(\lambda +p_{c}eb\right)}^{2}}$, $\frac{\beta^{m}}{\beta^{n}}=\frac{\bar{s}-bc-p_{c}eb-2\eta \delta_{s}}{\bar{s}-bc-p_{c}eb}$. Since $2bk-{\left(\lambda +p_{c}eb\right)}^{2}>0$ and $\bar{s}-bc-p_{c}eb>2\eta \delta_{s}$, we get $\beta^{m}<\beta^{n}<\beta^{*}$. Comparing $q^{*}$, $q^{n}$ and $q^{m}$, there are $q^{*}-q^{m}=\frac{2b^{2}k^{2}(\bar{s}-bc-p_{c}eb)-\eta \delta_{s}{\left[2bk-{\left(\lambda +p_{c}eb\right)}^{2}\right]}^{2}}{\left[2bk-{\left(\lambda +p_{c}eb\right)}^{2}]\times [4bk-{\left(\lambda +p_{c}eb\right)}^{2}\right]}$, $q^{m}-q^{n}=\frac{\eta \delta_{s}[2bk-{\left(\lambda +p_{c}eb\right)}^{2}]}{4bk-{\left(\lambda +p_{c}eb\right)}^{2}}$. Since $2bk-{\left(\lambda +p_{c}eb\right)}^{2}>0$, $\bar{s}-bc-p_{c}eb>2\eta \delta_{s}$, it is easy to obtain $q^{*}-q^{m}>0$, $q^{m}-q^{n}>0$, so $q^{n}<q^{m}<q^{*}$. □

Proposition 4 shows that to avoid their own risks, the manufacturer not only reduces the wholesale price of the product but also reduces the carbon emission reduction rate. By lowering the wholesale price of the products, the demand for them has increased.

**Proposition** **5.** *(I) The optimal product selling price $p^{n}$ in the case of risk-neutral and decentralized decisions is not only greater than the product selling price $p^{*}$ in the case of centralized decisions but also greater than the optimal product selling price $p^{m}$ in the case of risk-averse and decentralized decisions. (II) When $\lambda \leq p_{c}eb$, the selling price $p^{m}$ of the manufacturer in the case of a decentralized decision of risk aversion is greater than the selling price $p^{*}$ of the product in the case of a centralized decision. (III) When $\lambda >p_{c}eb$, let $\frac{2bk(\bar{s}-bc-p_{c}eb)[bk-\lambda (\lambda +p_{c}eb)]}{\delta_{s}[2bk-{\left(\lambda +p_{c}eb\right)}^{2}][2bk-(\lambda +p_{c}eb)(p_{c}eb-\lambda )]}=G_{1}$, and when $G_{1}>\eta$, the selling price of the manufacturer in the decentralized decision of risk aversion is greater than that in the centralized decision; when $G_{1}<\eta$, the selling price of the manufacturer in the decentralized decision of risk aversion is less than that in the centralized decision.*

**Proof.**  (I) By comparing $p^{*}$ and $p^{n}$ and $p^{n}$ and $p^{m}$, we can obtain: $p^{n}-p^{*}=\frac{-2bk}{4bk-{\left(\lambda +p_{c}eb\right)}^{2}}\times [p^{*}-\frac{\bar{s}}{b}]$, $p^{n}-p^{m}=\frac{\eta \delta_{s}[2bk-(\lambda +p_{c}eb)(p_{c}eb-\lambda )]}{b[4bk-{\left(\lambda +p_{c}eb\right)}^{2}]}$. Since $2bk-{\left(\lambda +p_{c}eb\right)}^{2}>0$ and assuming 1 $p^{*}<\frac{\bar{s}}{b}$, we can obtain $p^{n}>p^{*}$, $p^{n}>p^{m}$. (ii) Comparing $p^{*}$ and $p^{m}$, we can get: $p^{m}-p^{*}=\frac{2bk(\bar{s}-bc-p_{c}eb)[bk-\lambda (\lambda +p_{c}eb)]-\eta \delta_{s}[2bk-{\left(\lambda +p_{c}eb\right)}^{2}][2bk-(\lambda +p_{c}eb)(p_{c}eb-\lambda )]}{b[4bk-{\left(\lambda +p_{c}eb\right)}^{2}][2bk-{\left(\lambda +p_{c}eb\right)}^{2}]}$ Since $\bar{s}-bc-p_{c}eb>2\eta \delta_{s}$, we can conclude that $p^{m}-p^{*}>\frac{\left(\bar{s}-bc-p_{c}eb)(\lambda +p_{c}eb)(p_{c}eb-\lambda \right)}{b[2bk-{\left(\lambda +p_{c}eb\right)}^{2}]}$. Combined with $2bk-{\left(\lambda +p_{c}eb\right)}^{2}>0$, when $\lambda \leq p_{c}eb$, $p^{m}>p^{*}$. (iii) When $\lambda >p_{c}eb$, let $\frac{2bk(\bar{s}-bc-p_{c}eb)[bk-\lambda (\lambda +p_{c}eb)]}{\delta_{s}[2bk-{\left(\lambda +p_{c}eb\right)}^{2}][2bk-(\lambda +p_{c}eb)(p_{c}eb-\lambda )]}=G_{1}$, when $G_{1}-\eta >0$, it is easy to get $p^{m}>p^{*}$; when $G_{1}-\eta <0$, it is easy to get $p^{m}<p^{*}$. □

Proposition 5 shows that when the manufacturer is risk averse, the retailer will take the strategy of reducing the sales price to avoid its own risk, even lower than the centralized decision-making situation to increase sales.

**Proposition** **6.** *(i) When the manufacturer is risk averse, the retailer’s profit is larger than that in the risk-neutral case, and the manufacturer’s profit is smaller than that in the risk-neutral case. (ii) When ${\left[2bk-{\left(\lambda +p_{c}eb\right)}^{2}\right]}^{2}\leq 2bk{\left(\lambda +p_{c}eb\right)}^{2}$, the profit of supply chain system $\pi_{sc}^{m}$ under the risk aversion decentralized decision of the manufacturer is less than the profit $\pi_{sc}^{n}$ under the risk-neutral decentralized decision. (iii) When ${\left[2bk-{\left(\lambda +p_{c}eb\right)}^{2}\right]}^{2}>2bk{\left(\lambda +p_{c}eb\right)}^{2}$, let $\frac{4b^{2}k^{2}(\bar{s}-bc-p_{c}eb)}{\delta_{s}\{{\left[3bk-{\left(\lambda +p_{c}eb\right)}^{2}\right]}^{2}+3b^{2}k^{2}\}}=G_{2}$, and when $G_{2}<\eta$, the profit $\pi_{sc}^{m}$ of the supply chain.*

System in the case of the manufacturer’s risk-averse decentralized decision is greater than the profit $\pi_{sc}^{n}$ in the case of the risk-neutral decentralized decision. When $G_{2}>\eta$, the profit $\pi_{sc}^{m}$ of the supply chain system under the risk aversion decentralized decision is less than the profit $\pi_{sc}^{n}$ of the supply chain system under the risk-neutral decentralized decision. The profit of supply chain system $\pi_{sc}^{n}$ and profit of supply chain system $\pi_{sc}^{m}$ when the manufacturer makes a risk-neutral and decentralized decision are both smaller than those of supply chain system $\pi_{sc}^{*}$ when the manufacturer makes a centralized decision.

**Proof.**  (i) Comparing $\pi_{r}^{m}$ and $\pi_{r}^{n}$, $\pi_{m}^{m}$ and $\pi_{m}^{n}$, we can obtain: $\frac{\pi_{r}^{m}}{\pi_{r}^{n}}=\frac{{\left\{bk(\bar{s}-bc-p_{c}eb)+\eta \delta_{s}[2bk-{\left(\lambda +p_{c}eb\right)}^{2}]\right\}}^{2}}{{\left[bk(\bar{s}-bc-p_{c}eb)\right]}^{2}}$, $\frac{\pi_{m}^{m}-p_{c}A}{\pi_{m}^{n}-p_{c}A}=\frac{{\left(\bar{s}-bc-p_{c}eb-2\eta \delta_{s}\right)}^{2}}{{\left(\bar{s}-bc-p_{c}eb\right)}^{2}}$. Since $2bk-{\left(\lambda +p_{c}eb\right)}^{2}>0$ and $\bar{s}-bc-p_{c}eb>2\eta \delta_{s}$, it is easy to obtain: $\pi_{r}^{m}>\pi_{r}^{n}$, $\pi_{m}^{m}<\pi_{m}^{n}$. (ii) Comparing $\pi_{sc}^{m}$ and $\pi_{sc}^{n}$, we can get: $\pi_{sc}^{m}-\pi_{sc}^{n}=\frac{\eta \delta_{s}\{\eta \delta_{s}[12b^{2}k^{2}-6bk{\left(\lambda +p_{c}eb\right)}^{2}+{\left(\lambda +p_{c}eb\right)}^{4}]-4b^{2}k^{2}(\bar{s}-bc-p_{c}eb)\}}{b{\left[4bk-{\left(\lambda +p_{c}eb\right)}^{2}\right]}^{2}}$. Since $\bar{s}-bc-p_{c}eb>2\eta \delta_{s}$, we can get: $\pi_{sc}^{m}-\pi_{sc}^{n}<\frac{\eta^{2}\delta_{s}^{2}[4b^{2}k^{2}-6bk{\left(\lambda +p_{c}eb\right)}^{2}+{\left(\lambda +p_{c}eb\right)}^{4}]}{b{\left[4bk-{\left(\lambda +p_{c}eb\right)}^{2}\right]}^{2}}$. When ${\left[2bk-{\left(\lambda +p_{c}eb\right)}^{2}\right]}^{2}\leq 2bk{\left(\lambda +p_{c}eb\right)}^{2}$, $\pi_{sc}^{m}<\pi_{sc}^{n}$ is easily obtained. (iii) When ${\left[2bk-{\left(\lambda +p_{c}eb\right)}^{2}\right]}^{2}>2bk{\left(\lambda +p_{c}eb\right)}^{2}$, set $\frac{4b^{2}k^{2}(\bar{s}-bc-p_{c}eb)}{\delta_{s}\{{\left[3bk-{\left(\lambda +p_{c}eb\right)}^{2}\right]}^{2}+3b^{2}k^{2}\}}=G_{2}$, when $G_{2}<\eta$, it is easy to get $\pi_{sc}^{m}>\pi_{sc}^{n}$. When $G_{2}>\eta$, it is easy to get $\pi_{sc}^{m}<\pi_{sc}^{n}$. (iv) Comparing $\pi_{sc}^{*}$, $\pi_{sc}^{n}$ and $\pi_{sc}^{m}$, we can get: $\pi_{sc}^{*}-\pi_{sc}^{n}=\frac{2b^{2}k^{3}{\left(\bar{s}-bc-p_{c}eb\right)}^{2}}{[2bk-{\left(\lambda +p_{c}eb\right)}^{2}]{\left[4bk-{\left(\lambda +p_{c}eb\right)}^{2}\right]}^{2}}$, $\begin{array}{l} \pi_{sc}^{*}-\pi_{sc}^{m}=\frac{2b^{2}k^{3}{\left(\bar{s}-bc-p_{c}eb\right)}^{2}+4bk^{2}\eta \delta_{s}(\bar{s}-bc-p_{c}eb)[2bk-{\left(\lambda +p_{c}eb\right)}^{2}]}{[2bk-{\left(\lambda +p_{c}eb\right)}^{2}]{\left[4bk-{\left(\lambda +p_{c}eb\right)}^{2}\right]}^{2}}-\frac{\eta^{2}\delta_{s}^{2}}{b}\\ \;+\frac{2k\eta^{2}\delta_{s}^{2}[2bk-{\left(\lambda +p_{c}eb\right)}^{2}]}{{\left[4bk-{\left(\lambda +p_{c}eb\right)}^{2}\right]}^{2}} \end{array}$. From the previous analysis, we can see that there are $2bk-{\left(\lambda +p_{c}eb\right)}^{2}>0$ and $\bar{s}-bc-p_{c}eb>2\eta \delta_{s}$, so it is easy to obtain $\pi_{sc}^{*}-\pi_{sc}^{n}>0$, and at the same time, it can be inferred that $\pi_{sc}^{*}-\pi_{sc}^{m}>\frac{2k\eta^{2}\delta_{s}^{2}}{2bk-{\left(\lambda +p_{c}eb\right)}^{2}}-\frac{\eta^{2}\delta_{s}^{2}}{b}=\frac{\eta^{2}\delta_{s}^{2}{\left(\lambda +p_{c}eb\right)}^{2}}{b[2bk-{\left(\lambda +p_{c}eb\right)}^{2}]}>0$. □

Proposition 6 shows that the manufacturer’s risk-aversion coefficient reduces its own profit and increases the retailer’s profit, but the overall profit of the system is smaller than that of the centralized decision situation. This can be explained as the manufacturers sacrifice their own interests to increase the profits of their rivals to avoid their own profit risks.

**Property** **1.** *The optimal wholesale price $w^{m}$ and carbon emission reduction rate $\beta^{m}$ determined by the manufacturer are negatively correlated with the risk-aversion coefficient η when the manufacturer makes a decentralized decision on risk aversion. The optimal order quantity $q^{m}$ determined by the retailer is positively correlated with the risk-aversion coefficient η, while the optimal selling price $p^{m}$ is negatively correlated with the risk-aversion coefficient η.*

**Proof.**  Derivation of $w^{m}$ and $\beta^{m}$ to the risk-aversion coefficient η, respectively: $\frac{\partial w^{m}}{\partial \eta }=\frac{-\delta_{s}[4k-(\lambda +p_{c}eb)p_{c}e]}{4bk-{\left(\lambda +p_{c}eb\right)}^{2}}$, $\frac{\partial \beta^{m}}{\partial \eta }=\frac{-2\delta_{s}(\lambda +p_{c}eb)}{4bk-{\left(\lambda +p_{c}eb\right)}^{2}}$. Since $2bk-{\left(\lambda +p_{c}eb\right)}^{2}>0$, so $4k-(\lambda +p_{c}eb)p_{c}e>0$, then $\partial w^{m}/\partial \eta <0$ and $\partial \beta^{m}/\partial \eta <0$. Deriving $q^{m}$ and $p^{m}$ to the risk-aversion coefficient η respectively: $\frac{\partial q^{m}}{\partial \eta }=\frac{\delta_{s}[2bk-{\left(\lambda +p_{c}eb\right)}^{2}]}{4bk-{\left(\lambda +p_{c}eb\right)}^{2}}$, $\frac{\partial p^{m}}{\partial \eta }=\frac{-\delta_{s}[2bk-(\lambda +p_{c}eb)(p_{c}eb-\lambda )]}{b[4bk-{\left(\lambda +p_{c}eb\right)}^{2}]}$. Since $2bk-{\left(\lambda +p_{c}eb\right)}^{2}>0$, it is easy to get $2bk-(\lambda +p_{c}eb)(p_{c}eb-\lambda )>0$, then: $\partial q^{m}/\partial \eta >0$, $\partial p^{m}/\partial \eta <0$.

Property 1 shows that when the manufacturer pays attention to risk avoidance, to meet its own risk avoidance needs, it not only reduces the wholesale price of the product but also reduces the carbon emission reduction rate. As wholesale prices have fallen, retailers have increased orders and lowered prices.

**Property** **2.** *The retailer’s expected utility $U(\pi_{r}^{m})$ is positively correlated with the risk-aversion coefficient η, while the manufacturer’s expected utility $U(\pi_{m}^{m})$ is negatively correlated with the risk-aversion coefficient η in the manufacturer’s risk-aversion diversification decision.*

**Proof.**  Derivation of $U(\pi_{r}^{m})$ and $U(\pi_{m}^{m})$ to the risk-aversion coefficient η, respectively: $\frac{\partial U(\pi_{r}^{m})}{\partial \eta }=\frac{2\delta_{s}[2bk-{\left(\lambda +p_{c}eb\right)}^{2}]\{bk(\bar{s}-bc-p_{c}eb)+\eta \delta_{s}[2bk-{\left(\lambda +p_{c}eb\right)}^{2}]\}}{b{\left[4bk-{\left(\lambda +p_{c}eb\right)}^{2}\right]}^{2}}$, $\frac{\partial U(\pi_{m}^{m})}{\partial \eta }=\frac{-4k\delta_{s}(\bar{s}-bc-p_{c}eb-2\eta \delta_{s})}{2[4bk-{\left(\lambda +p_{c}eb\right)}^{2}]}$. Since $2bk-{\left(\lambda +p_{c}eb\right)}^{2}>0$ and $\bar{s}-bc-p_{c}eb>2\eta \delta_{s}$, then $\partial U(\pi_{r}^{m})/\partial \eta >0$ and $\partial U(\pi_{m}^{m})/\partial \eta <0$. □

Property 2 shows that when the manufacturer pays attention to risk aversion, to avoid risk, the manufacturer will not hesitate to reduce the wholesale price to increase the order quantity of the product. Therefore, the expected utility of the manufacturer decreases with the increase of the risk-aversion coefficient, and the expected utility of the retailer increases with the increase of the risk-aversion coefficient.

### 3.3. Contract Coordination Decision Models under Different Circumstances Are Built and Solved

As consumers have a low carbon preference, manufacturers will increase the production of low-carbon products to expand the market demand, but this will lead to an increase in the initial capital investment of manufacturers. To increase the enthusiasm of manufacturers, retailers will choose to take the initiative to bear part of the cost of low-carbon emission reduction to achieve a win–win situation. When the manufacturer and the retailer reach a cost-sharing contract for low carbon emission reduction, the proportion of the retailer to bear the investment cost of carbon emission reduction is ϕ, and the proportion of the manufacturer to bear is (1−ϕ). At this point, the expected profit functions of manufacturers and retailers are:

(24) $$E(\pi_{rn}^{c})=(p-w)(\bar{s}-bp+\lambda \beta )-\frac{1}{2}\phi k\beta^{2}$$

(25) $$E(\pi_{mn}^{c})=[w-c](\bar{s}-bp+\lambda \beta )+p_{c}[A-e(1-\beta )(\bar{s}-bp+\lambda \beta )]-\frac{1}{2}(1-\phi )k\beta^{2}$$

#### 3.3.1. Risk-Neutral Coordination Decision-Making Process of Manufacturers

Furthermore, we continue with the backward derivation. Retailers act as followers to determine the optimal unit price based on the manufacturer’s decision. From Equations (24) and (25), we can obtain the following results.

**Proposition** **7.** *When $4bk(1-\phi )-{\left(\lambda +p_{c}eb\right)}^{2}>0$, the manufacturer’s risk-neutral coordinated decision model has a unique product wholesale price w, emission reduction rate per unit product β, and product sales price p to maximize the expected profit of manufacturers and retailers. At this point, the optimal wholesale price of product $w_{n}^{c}$, emission reduction rate of unit product $\beta_{n}^{c}$, product sales price $p_{n}^{c}$ and market demand $q_{n}^{c}$ can be, respectively expressed as:*

(26) $$w_{n}^{c}=\frac{2k(1-\phi )(\bar{s}+bc+p_{c}eb)-(\lambda +p_{c}eb)(p_{c}es+p_{c}e\lambda +c\lambda )}{4bk(1-\phi )-{\left(\lambda +p_{c}eb\right)}^{2}}$$

(27) $$\beta_{n}^{c}=\frac{\left(\bar{s}-bc-p_{c}eb)(\lambda +p_{c}eb\right)}{4bk(1-\phi )-{\left(\lambda +p_{c}eb\right)}^{2}}$$

(28) $$p_{n}^{c}=\frac{k(1-\phi )(3\bar{s}+bc+p_{c}eb)-(\lambda +p_{c}eb)(p_{c}e\bar{s}+p_{c}e\lambda +c\lambda )}{4bk(1-\phi )-{\left(\lambda +p_{c}eb\right)}^{2}}$$

(29) $$q_{n}^{c}=\frac{bk(1-\phi )(\bar{s}-bc-p_{c}eb)}{4bk(1-\phi )-{\left(\lambda +p_{c}eb\right)}^{2}}$$

The proof of Proposition 7 is given in Appendix D.

Proposition 7 shows that when manufacturers make risk-neutral coordinated decisions, the appropriate value of contract parameters can ensure the existence and uniqueness of supply chain members’ decisions. To make all members of the supply chain take the initiative to accept the contract, in the following part, we will give the specific value range of the cost-sharing coefficient ϕ to realize the Pareto improvement of the supply chain system and give the optimal cost-sharing coefficient value determined by the retailer.

By subbing $w_{n}^{c}$, $\beta_{n}^{c}$ and $p_{n}^{c}$ into $E(\pi_{rn}^{c})$ and $E(\pi_{mn}^{c})$, the expected profit function of retailers and manufacturers in the manufacturer’s risk-neutral contract coordination decision can be obtained. Furthermore, the expected profit function of the whole system can be obtained as follows:

(30) $$E(\pi_{rn}^{c})=\frac{k{\left(\bar{s}-bc-p_{c}eb\right)}^{2}[2bk{\left(1-\phi \right)}^{2}-\phi {\left(\lambda +p_{c}eb\right)}^{2}]}{2{\left[4bk(1-\phi )-{\left(\lambda +p_{c}eb\right)}^{2}\right]}^{2}}$$

(31) $$E(\pi_{mn}^{c})=\frac{k(1-\phi ){\left(\bar{s}-bc-p_{c}eb\right)}^{2}}{2\left[4bk(1-\phi )-{\left(\lambda +p_{c}eb\right)}^{2}\right]}+p_{c}A$$

(32) $$E(\pi_{scn}^{c})=E(\pi_{rn}^{c})+E(\pi_{mn}^{c})=\frac{k{\left(\bar{s}-bc-p_{c}eb\right)}^{2}[6bk{\left(1-\phi \right)}^{2}-{\left(\lambda +p_{c}eb\right)}^{2}]}{2{\left[4bk(1-\phi )-{\left(\lambda +p_{c}eb\right)}^{2}\right]}^{2}}+p_{c}A$$

#### 3.3.2. Manufacturers’ Risk Avoidance Coordination Decision-Making Process

Similar to Section 3.2.3, the manufacturer’s risk-aversion coefficient η>0 is introduced, and the utility function of the manufacturer and the retailer can be expressed as:

(33) $$U(\pi_{mm}^{c})=E(\pi_{mn}^{c})-\eta \sqrt{Var(\pi_{mn}^{c})}=[w-c-p_{c}e(1-\beta )](\bar{s}-bp+\lambda \beta )+p_{c}A-\frac{1}{2}(1-\phi )k\beta^{2}-\eta \delta_{s}[w-c-p_{c}e(1-\beta )]$$

(34) $$U(\pi_{rm}^{c})=E(\pi_{rn}^{c})=(p-w)(\bar{s}-bp+\lambda \beta )-\frac{1}{2}\phi k\beta^{2}$$

Through Equations (33) and (34), we can obtain the following results.

**Proposition** **8.** *When $4bk(1-\phi )-{\left(\lambda +p_{c}eb\right)}^{2}>0$, the manufacturer risk-aversion coordinated decision model has a unique product wholesale price w, emission reduction rate per unit product β, and product sales price p, which maximizes the expected profit of manufacturers and retailers. At this point, the optimal wholesale price of product $w_{m}^{c}$, emission reduction rate of unit product $\beta_{m}^{c}$, product sales price $p_{m}^{c}$ and market demand $q_{m}^{c}$ can be, respectively expressed as:*

(35) $$w_{m}^{c}=\frac{2k(1-\phi )(\bar{s}+bc+p_{c}eb-2\eta \delta_{s})-(\lambda +p_{c}eb)(p_{c}e\bar{s}+p_{c}e\lambda +c\lambda -2\eta \delta_{s}p_{c}e)}{4bk(1-\phi )-{\left(\lambda +p_{c}eb\right)}^{2}}$$

(36) $$\beta_{m}^{c}=\frac{\left(\bar{s}-bc-p_{c}eb-2\eta \delta_{s})(\lambda +p_{c}eb\right)}{4bk(1-\phi )-{\left(\lambda +p_{c}eb\right)}^{2}}$$

(37) $$p_{m}^{c}=\frac{bk(1-\phi )(3\bar{s}+bc+p_{c}eb-2\eta \delta_{s})-(\lambda +p_{c}eb)(p_{c}eb\bar{s}+p_{c}eb\lambda +cb\lambda -p_{c}eb\eta \delta_{s}+\eta \delta_{s}\lambda )}{b[4bk(1-\phi )-{\left(\lambda +p_{c}eb\right)}^{2}]}$$

(38) $$q_{m}^{c}=\frac{bk(1-\phi )(\bar{s}-bc-p_{c}eb)+\eta \delta_{s}[2bk(1-\phi )-{\left(\lambda +p_{c}eb\right)}^{2}]}{4bk(1-\phi )-{\left(\lambda +p_{c}eb\right)}^{2}}$$

The proof of Proposition 8 is given in Appendix E.

Proposition 8 shows that when manufacturers make coordinated decisions on risk aversion, the appropriate value of contract parameters can ensure the existence and uniqueness of supply chain members’ decisions. In the next section, we will give the specific value range of the cost-sharing coefficient ϕ.

Let bk=B, ${\left(\lambda +p_{c}eb\right)}^{2}=C$, $(\bar{s}-bc-p_{c}eb)=S$, $\eta \delta_{s}=H$, and substitute $w_{m}^{c}$, $\beta_{m}^{c}$ and $p_{m}^{c}$ into $U(\pi_{rm}^{c})$ and $U(\pi_{mm}^{c})$ to obtain the expected profit function of retailers and manufacturers in the coordinated decision of the manufacturer’s risk avoidance contract. Furthermore, the expected profit function of the whole system can be obtained as follows:

(39) $$U(\pi_{rm}^{c})=\frac{2{\left[B(1-\phi )(S+2H)-HC\right]}^{2}-\phi BC{\left(S-2H\right)}^{2}}{2b{\left[4B(1-\phi )-C\right]}^{2}}$$

(40) $$U(\pi_{mm}^{c})=\frac{k(1-\phi ){\left(S-2H\right)}^{2}}{2[4B(1-\phi )-C]}+p_{c}A$$

(41) $$U(\pi_{scm}^{c})=U(\pi_{rm}^{c})+U(\pi_{mm}^{c})=\frac{2{\left[B(1-\phi )(S+2H)-HC\right]}^{2}+B{\left(S-2H\right)}^{2}[4B{\left(1-\phi \right)}^{2}-C]}{2b{\left[4B(1-\phi )-C\right]}^{2}}+p_{c}A$$

**Proposition** **9.** Under the manufacturer’s risk-neutral decision, (I) when the coefficient meets: $0<\phi <\frac{{\left(\lambda +p_{c}eb\right)}^{2}[4bk-{\left(\lambda +p_{c}eb\right)}^{2}]}{2bk[8bk-{\left(\lambda +p_{c}eb\right)}^{2}]}⊗$, the carbon emission reduction cost-sharing contract can realize the Pareto improvement of supply chain members’ profits. (II) When the coefficient meets: $\frac{5{\left(\lambda +p_{c}eb\right)}^{2}-8bk}{16bk}<\phi <\frac{{\left(\lambda +p_{c}eb\right)}^{2}[4bk-{\left(\lambda +p_{c}eb\right)}^{2}]}{2bk[8bk-{\left(\lambda +p_{c}eb\right)}^{2}]}$, the optimal cost-sharing coefficient determined by the retailer is $\phi^{*}=\frac{{\left(\lambda +p_{c}eb\right)}^{2}}{8bk}$. (III) When $0<\phi \leq \frac{5{\left(\lambda +p_{c}eb\right)}^{2}-8bk}{16bk}$, the retailer’s profit function $\pi_{rn}^{c}$ is a lower convex function of ϕ, and the smaller $\phi^{*}$ is, the better.

The proof of Proposition 9 is given in Appendix F.

**Proposition** **10.** Under the manufacturer’s risk-aversion decision, if bk=B, ${\left(\lambda +p_{c}eb\right)}^{2}=C$, $(\bar{s}-bc-p_{c}eb)=S$, $\eta \delta_{s}=H$, (I) when $0<\phi <\frac{\left(4B-C)[CS+(16B-6C)H\right]}{2B[(8B-C)S+(16B-6C)H]}$, the carbon emission reduction cost-sharing contract can realize the Pareto improvement of supply chain members’ profits. (II) When the cost-sharing coefficient of carbon emission reduction meets $\frac{5CS+3H(16B-6C)-8BS}{16BS}<\phi <\frac{\left(4B-C)[CS+(16B-6C)H\right]}{2B[(8B-C)S+(16B-6C)H]}$, the optimal cost-sharing coefficient determined by the retailer is $\phi^{*}=\frac{CS+(16B-6C)H}{8BS}$; (III) When $0<\phi \leq \frac{5CS+3H(16B-6C)-8BS}{16BS}$, retailer profit function $\pi_{rm}^{c}$ is a lower convex function of ϕ, and the smaller $\phi^{*}$ is, the better.

The proof of Proposition 10 is given in Appendix G.

Propositions 9 and 10 give the optimal cost-sharing ratio of carbon emission reduction investment undertaken by retailers when the manufacturer makes a risk-neutral and risk-averse diversification decision, respectively. The above carbon emission reduction investment cost contract makes retailers and manufacturers expect higher profits than before the contract. On the other hand, the contract cannot realize the perfect coordination of the supply chain system but can only realize the Pareto improvement of the supply chain members’ profits.

## 4. Numerical Analysis

Similar to Bai et al. [17], we discuss the impact of risk aversion on low-carbon supply chain decision-making through numerical analysis. Suppose that a low-carbon supply chain is composed of multiple manufacturers and retailers, among which the manufacturer is responsible for the production of low-carbon products of the same quality, and the retailer is responsible for the sale of low-carbon products. To facilitate the solution, price competition between manufacturers and retailers at the same level is not considered, and the average values of relevant parameters obtained through the survey are as follows:$\bar{s}=400$, $\delta_{s}=5$, b=5, c=4, k=500, $p_{c}=1$, e=2, λ=1, A=500.

### 4.1. Results Analysis

In order to verify the feasibility of the risk-avoidance decision model established in this paper, we take the manufacturer’s risk-avoidance coefficient η=5 and substitute it with other parameters into the centralized decision model and decentralized decision model. The values of the decision variables are shown in Table 2.

It can be seen from Table 2 that manufacturers’ risk-avoidance behavior has a great impact on the decision-making results of the system. Compared with decentralized decision-making, the manufacturer’s risk-aversion preference reduces its own profit but increases its opponent’s profit, and also reduces the emission reduction rate of the system. Obviously, the decision of manufacturers to sacrifice their own interests to avoid risks is not conducive to the enterprise’s economic and environmental objectives. In order to explore the influence rules of risk avoidance coefficient, cost-sharing coefficient and consumers’ low-carbon preference coefficient on the optimal decision-making of the system, sensitivity analysis of each parameter will be conducted later.

### 4.2. Impact of Manufacturers’ Risk Aversion on Pricing Decisions

**Figure 1:**
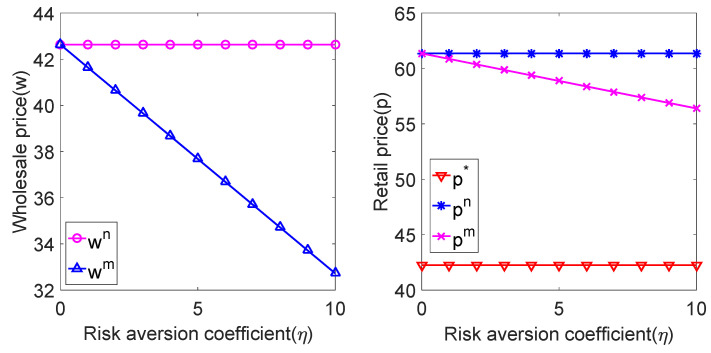
Relationship among w, p and η.

Substituting the above parameters into the expressions of wholesale price and sales price under centralized decision model and decentralized decision model, the relationship between wholesale price and sales price and risk-aversion coefficient η can be obtained, as shown in Figure 1:

As seen from Figure 1, when the manufacturer has the risk-aversion characteristic, the wholesale price of the product is lower than the risk-neutral situation, and it decreases with the increase of the manufacturer’s risk-aversion coefficient. The product selling price is higher than the centralized decision-making situation, lower than the risk-neutral decentralized decision-making situation and decreases with the increase of the manufacturer’s risk-aversion coefficient. In other words, when manufacturers focus on risk aversion, to avoid their own risks and improve product sales in the market, manufacturers adopt the strategy of reducing wholesale prices, while retailers adopt the strategy of reducing sales prices.

**Figure 2:**
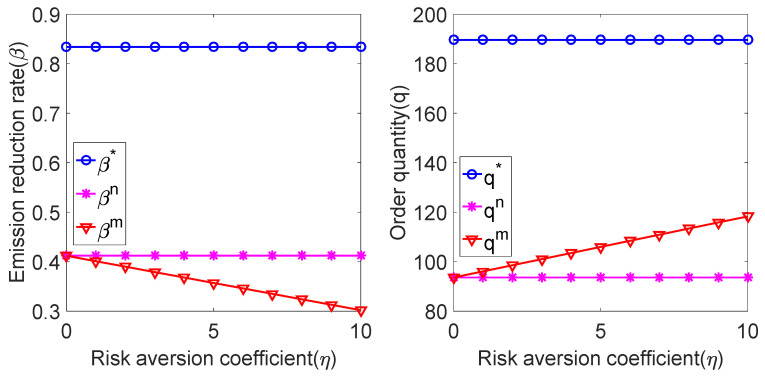
Relationship among β, q and η.

We substituted the above parameters into the expressions of the carbon emission reduction rate and product order quantity under centralized decision model and decentralized decision model to obtain the relationship between the carbon emission reduction rate and product order quantity and the risk-aversion coefficient η. As shown in Figure 2:

As shown in Figure 2, the carbon emission reduction rate in the risk-neutral case is lower than that in the centralized decision-making case, the carbon emission reduction rate in the risk-averse case is lower than that in the risk-neutral case, and it decreases with the increase in the manufacturer’s risk-aversion coefficient. The product order quantity in the risk-aversion condition is higher than that in the risk-neutral condition and lower than that in the centralized decision-making condition, and it increases with the increase in the risk-aversion coefficient. Therefore, when manufacturers pay attention to risk aversion, to avoid their own risks, manufacturers will not only reduce the carbon emission reduction rate but also reduce the wholesale price of products, thus increasing market demand.

Obviously, this strategy is not only inconsistent with the carbon emission reduction target of the system, but also is not conducive to the establishment of an enterprise brand. In order to alleviate manufacturers’ risk anxiety, the government can encourage them to improve their carbon emission reduction rate through carbon subsidies, and relevant enterprises can also share the cost of carbon emission reduction through cooperation contracts. These measures will benefit the sustainable development of supply chain related enterprises.

### 4.3. Impact of Manufacturers’ Risk Aversion on Supply Chain Profits

**Figure 3:**
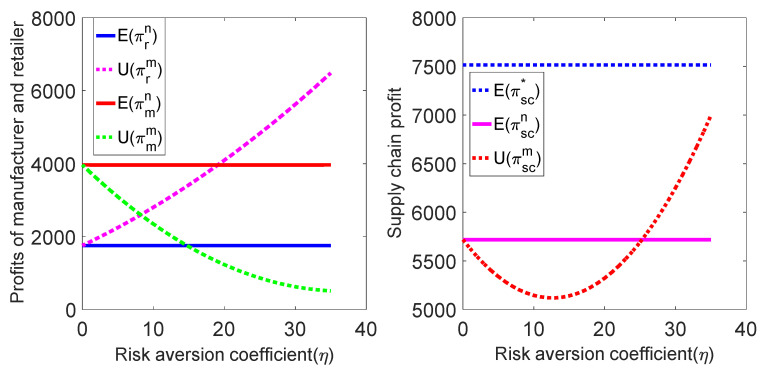
Relationship among π, $\pi_{sc}$ and η.

Substituting the above parameters into the profit expressions of retailers, manufacturers and supply chains, the relationship between $\pi_{r}$, $\pi_{m}$, $\pi_{sc}$ and the risk-aversion coefficient η can be obtained under centralized decision model and decentralized decision model, as shown in Figure 3:

As seen from Figure 3, when the manufacturer has the risk-aversion characteristic, the profit of the manufacturer is less than that of the fair neutral situation, and it decreases with the increase of the manufacturer’s risk-aversion coefficient η. The retailer’s profit is larger than that in the fair neutral case and increases with the increase in the manufacturer’s risk-aversion coefficient η. If consumers have a low carbon preference, the profit of the supply chain system will first decrease with the increase of the manufacturer’s risk-aversion coefficient η. When η reaches a certain value, the profit of the supply chain system increases with the increase of the manufacturer’s risk-aversion coefficient η and even appears to be greater than the risk-neutral situation. At the same time, the profit of the supply chain system under risk aversion and risk neutrality is smaller than that under centralized decision making.

This result indicates that when manufacturers focus on risk avoidance, they sacrifice part of their own interests in order to avoid their own risks, which increases the part of their rivals’ profits. In contrast, manufacturer’s profit gradually decreases with the increase of risk-aversion coefficient η, while retailer’s profit increases rapidly with the increase of risk-aversion coefficient η.

### 4.4. Influence of the Cost-Sharing Coefficient on Supply Chain Profits

**Figure 4:**
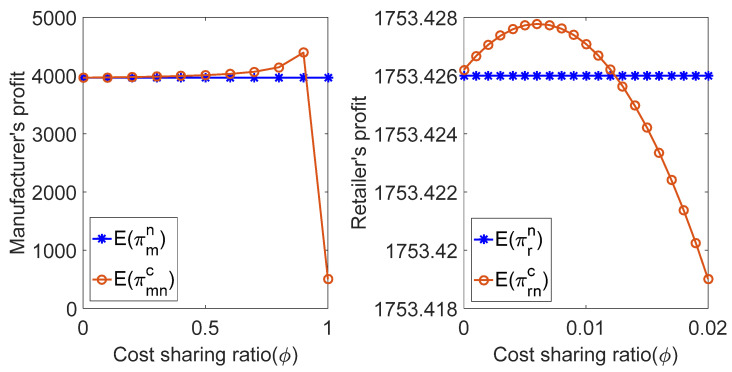
Comparison of profit of supply chain members under risk-neutral decision.

**Figure 5:**
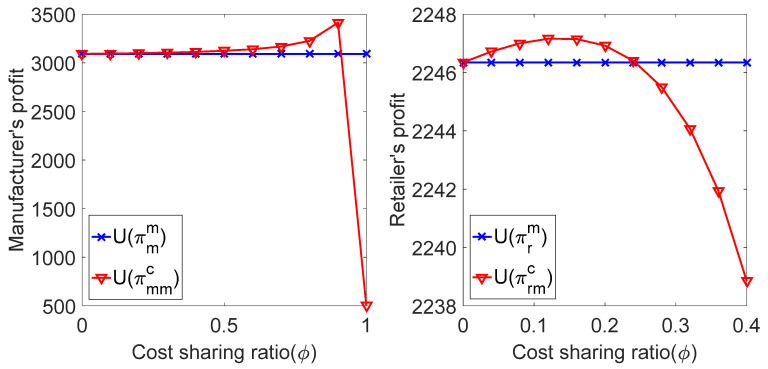
Comparison of the profits of supply chain members under risk-aversion decisions.

We substituted the above parameters into the profit expression of retailers and manufacturers in the coordination decision of the cost-sharing contract, and the relationship between $\pi_{r}$, $\pi_{m}$ and the cost-sharing coefficient ϕ in the coordination decision mode of the contract was obtained, as shown in Figure 4 and Figure 5.

As shown in in Figure 4 and Figure 5, when the manufacturer makes a risk-neutral decision and a risk-averse decision, the profit of the manufacturer and the retailer first increases with the increase in the cost-sharing coefficient ϕ. When ϕ reaches a certain value, it decreases with the increase in the cost-sharing coefficient ϕ. In the increase period, the profit of both the manufacturer and retailer under the cost-sharing contract is higher than that under the decentralized decision. This indicates that the cost-sharing contract can achieve Pareto improvement of supply chain members’ profits when parameter ϕ is an appropriate value, regardless of the manufacturer’s risk-neutral decision or risk-aversion decision.

### 4.5. Impact of Consumers’ Low-Carbon Preference on Supply Chain Profits

To verify the results of propositions 3 and 4 and improve the low-carbon preference coefficient of consumers, let λ=35($\lambda >p_{c}eb$, ${\left[2bk-{\left(\lambda +p_{c}eb\right)}^{2}\right]}^{2}<2bk{\left(\lambda +p_{c}eb\right)}^{2}$).

**Figure 6:**
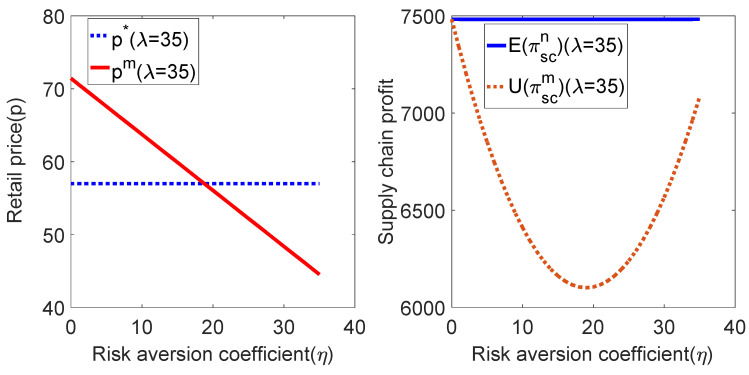
Relationship among p, $\pi_{sc}$ and η.

Then, the above parameters are substituted into the expressions of the sales price and profit of the supply chain system under different decision modes, and the relationship between the sales price and profit of the supply chain system and the risk-aversion coefficient η can be obtained, as shown in Figure 6:

As shown in Figure 6, when the manufacturer has the risk-aversion characteristic, if the consumer’s low-carbon preference coefficient λ is high, the selling price will decrease with the increase of the risk-aversion coefficient η. When η reaches a certain value, the selling price under risk aversion is lower than the selling price under centralized decision-making. The profit of the supply chain system first decreases with the increase of the risk-aversion coefficient η and then increases with the increase of the risk-aversion coefficient η, but the profit of the system under the risk aversion is always lower than that under the decentralized decision. It can be understood that when consumers have the high preference for low-carbon products, manufacturers must increase the input of carbon emission reduction costs to meet emission-sensitive demands and improve the market share of high-carbon products. As the risk-aversion factor η continues to increase, manufacturers will decide whether to expand investment in emission reduction technologies. At this time, to gain more profits, retailers have to gradually reduce the sales price of products, which ultimately reduces the profit of the supply chain system. Since a decrease in the selling price will increase market demand, when the selling price drops to a certain value, the profit of the supply chain system will rise again.

In conclusion, considering the carbon quota policy, this paper analyzes the impact of the manufacturer’s risk-aversion characteristics and retailer’s low carbon preference on supply chain pricing decisions and designs a Pareto improvement of the cost-sharing contract implementation system. Our results provide insights into the relationship between risk-aversion characteristics and different prices and carbon emission rates. It also helps managers and decision makers choose the most effective low-carbon and price strategies in the face of risk-aversion characteristics and adopt appropriate contract coordination schemes to improve the overall system returns.

## 5. Conclusions

In this paper, the mean-variance method and Stackelberg game theory are used to study the decision-making problem of a low-carbon supply chain dominated by risk-averse manufacturers under a carbon quota policy. In general, we comprehensively compare and analyze the relationship between risk aversion and various parameters and system profit under risk neutrality and focus on the impact of risk-aversion factors on relevant parameters and system profit. Furthermore, a cost-sharing contract is proposed, and the decision-making process of supply chain coordination under different circumstances is studied.

The results show that if consumers have a high preference for low-carbon products, the manufacturer’s risk-aversion coefficient will lead to a lower selling price than the centralized decision, and the profit of the supply chain system will be further reduced. Therefore, the level of retailers’ low-carbon preference has a direct impact on manufacturers’ investment in carbon emission reduction costs. When retailers’ low-carbon preference is high, although manufacturers increase their investment in carbon emission reduction costs, risk aversion should not be considered. At the same time, when the cost-sharing contract is adopted for coordination, Pareto improvement of the profits of supply chain members can be achieved when the parameters of the cost-sharing contract are appropriate, regardless of the manufacturer’s risk-neutral decision or risk-aversion decision. Our analysis shows that cost-sharing contracts result in higher emission reduction rates and profitability for manufacturers and retailers. In this case, the manufacturer and the retailer, through the negotiation to determine the cost-sharing factor, can lead to the ideal situation. On the other hand, from the perspective of risk aversion, manufacturers have harmed their own interests because of risk-aversion behavior, while retailers have gained profits. When the manufacturer has the risk-aversion characteristic, it will try to reduce the emission reduction investment cost and wholesale price to avoid the risk. In this case, the retailer will increase the number of orders and ultimately make a profit for itself.

This study is of great significance to guide the sustainable development of the supply chain. However, as rational economic man, enterprises have the tendency of risk aversion. However, in a low-carbon supply chain, manufacturers’ risk-aversion behavior not only reduces the emission reduction rate but also damages their own profits. Therefore, in the low-carbon supply chain dominated by upstream nodal enterprises, to improve their own profits and generate higher environmental benefits, the leading enterprises should make decisions with a risk-neutral attitude. On the other hand, with the increase in consumers’ preference for low carbon, manufacturers need to increase the input of emission reduction costs to meet market demand. To reduce the increased risk concerns of manufacturers due to increased emission reduction costs, governments can use subsidies to increase the incentive of manufacturers to reduce emissions. From the point of view of contract selection, a cost-sharing contract can ensure a win–win situation between the manufacturer and retailer. To compensate manufacturers for the increased cost of adopting low-carbon technologies, manufacturers can negotiate from the perspective of the green quality of products to ensure their own interests, which not only reduces carbon emissions and protects the environment but also improves the enthusiasm of supply chain participants. The results of this paper are conducive to understanding the relationship among risk aversion, the carbon emission reduction rate, the product price and the cost-sharing coefficient, helping decision makers choose an effective pricing strategy and coordination mechanism, and guiding enterprises in how to invest in low-carbon technology.

The contributions of this paper are as follows: (1) We use the mean variance method to describe the manufacturer’s risk-aversion characteristics, incorporate carbon allowance policies and risk-aversion factors into a two-level supply chain led by manufacturers with low-carbon technology investment, and then use game theory methods to determine the best carbon reduction ranking rate, sales price, wholesale price, and profit of system members. (2) To reflect the cooperation between the manufacturer and the retailer, the cost-sharing contract is further proposed by using the coordination conditions of the supply chain system, and the optimal value of the cost-sharing coefficient and other decision variables is obtained by using the method in (1). (3) This study further analyzes the impact of manufacturer risk aversion on decentralized decision making and cost-sharing contract collaboration. We obtain the optimal decision variable values, member profit values and contract parameter values related to the risk-aversion coefficient. Moreover, we study the impact of risk-aversion characteristics on supply chain members and system decisions through strict numerical analysis. (4) By comparing the risk aversion and risk-neutral decision-making of manufacturers, some interesting insights are obtained.

It should be noted that this study has some limitations. First, the article does not consider the risk-aversion characteristics of retailers, but in fact, both manufacturers and retailers have risk-aversion characteristics. Second, the cost-sharing contract adopted in this paper can only achieve Pareto improvement of the supply chain system but cannot achieve perfect coordination. Third, this paper only considers the carbon quota policy for research, which is inconsistent with the emission reduction regulations of some regions or countries. Moreover, taking the carbon quota as an endogenous variable for decision-making is more conducive to the operation and management of enterprises. These provide directions for future research.

## Figures and Tables

**Table 1:** Parameters and decision variables.

	Parameter
c	manufacturer’s production cost of per unit of product
q	product demand function
e	manufacturer’s initial carbon footprint per unit
A	free carbon quotas allocated by the government
$p_{c}$	unit carbon trading price
η	manufacturer’s risk aversion factor
s	product market capacity. Where, s follows a normal distribution with mean value $\bar{s}$ and standard deviation $\delta_{s}$.
b	sales price sensitivity coefficient, and satisfy $\bar{s}-bp>0$
λ	consumer low-carbon preference coefficient
k	carbon emission reduction investment cost coefficient
$E(\pi_{r})$	retailer’s expected profit function
$E(\pi_{m})$	manufacturer’s expected profit function
$E(\pi_{sc})$	the expected profit function of supply chain
$U(\pi_{r})$	retailer’s utility function
$U(\pi_{m})$	manufacturer’s utility function
$U(\pi_{sc})$	utility function of supply chain
$Y^{*}$	the value of Y in the centralized decision case, $Y\in \{w,p,\beta ,\pi_{r},\pi_{m},\pi_{sc}\}$
$Y^{n}$	the value of Y in the risk-neutral decentralized decision case,$Y\in \{w,p,\beta ,\pi_{r},\pi_{m},\pi_{sc}\}$
$Y^{m}$	the value of Y in the manufacturer’s risk-aversion decision,$Y\in \{w,p,\beta ,\pi_{r},\pi_{m},\pi_{sc}\}$
$Y^{c}$	the value of Y in the case of contract coordination decision, $Y\in \{w,p,\beta ,\pi_{r},\pi_{m},\pi_{sc}\}$
	**Decision variables**
w	the wholesale price per unit of product provided by the manufacturer
p	retailer’s sales price per unit of product
β	emission reduction rate per unit product

**Table 2:** Comparison of decision results under different modes.

Variables	Centralized Decision-Making	Decentralized Decision-Making (Risk Neutral)	Decentralized Decision-Making (Risk Aversion) (η=5)
q	189.588	93.633	105.978
p	42.249	61.356	58.881
β	0.834	0.412	0.357
w		42.630	37.680
$\pi_{r}$		1753.426	2246.346
$\pi_{m}$		3964.419	3091.355
$\pi_{sc}$	7514.757	5717.845	5337.701

## Data Availability

Not applicable.

## Appendix A

**Proof** **of** **Proposition** **1.** The Hessian matrix $H_{1}(p,\beta )$ of $E(\pi_{sc}^{*})$ regarding p and β can be expressed as $H_{1}(p,\beta )=\left[\begin{array}{cc} -2b & \lambda -p_{c}eb\\ \lambda -p_{c}eb & 2p_{c}e\lambda -k \end{array}\right]$. Because $D_{1}=-2b<0$, when $D_{2}=2bk-{\left(\lambda +p_{c}eb\right)}^{2}>0$, $H_{1}(p,\beta )$ is a negative definite matrix, and $E(\pi_{sc}^{*})$ is a concave function of p and β. Setting $\partial E(\pi_{sc}^{*})/\partial p=0$, $\partial E(\pi_{sc}^{*})/\partial \beta =0$, and parallel to the equations, $p^{*}$ and $\beta^{*}$ can be obtained: $p^{*}=\frac{k(\bar{s}+bc+p_{c}eb)-(\lambda +p_{c}eb)(p_{c}e\bar{s}+p_{c}e\lambda +c\lambda )}{2bk-{\left(\lambda +p_{c}eb\right)}^{2}}$, $\beta^{*}=\frac{\left(\bar{s}-bc-p_{c}eb)(\lambda +p_{c}eb\right)}{2bk-{\left(\lambda +p_{c}eb\right)}^{2}}$. Substituting $p^{*}$ and $\beta^{*}$ into the expressions of q and $E(\pi_{sc}^{*})$, the order quantity and the expected profit of the whole supply chain under the centralized control decision can be obtained as follows: $q^{*}=\frac{bk(\bar{s}-bc-p_{c}eb)}{2bk-{\left(\lambda +p_{c}eb\right)}^{2}}$, $E(\pi_{sc}^{*})=\frac{k{\left(\bar{s}-bc-p_{c}eb\right)}^{2}}{2[2bk-{\left(\lambda +p_{c}eb\right)}^{2}]}+p_{c}A$. To ensure that the variable is not negative, it must also satisfy. $\bar{s}-bc-p_{c}eb>0$ and $k(\bar{s}+bc+p_{c}eb)-(\lambda +p_{c}eb)(p_{c}e\bar{s}+p_{c}e\lambda +c\lambda )>0$. □

## Appendix B

**Proof** **of** **Proposition** **2.** Since $\partial^{2}E(\pi_{r}^{n})/\partial p^{2}=-2b<0$, let $\partial E(\pi_{r}^{n})/\partial p=0$; then, the reaction function of p on w and β can be obtained: $p^{n}=(\bar{s}+\lambda \beta +bw)/2b$. Substituting Equation $p^{n}$ into $E(\pi_{m}^{n})$, the Hessian matrix $H_{2}(w,\beta )$ of $E(\pi_{m}^{n})$ concerning w and β can be expressed as $H_{2}(w,\beta )=\left[\begin{array}{cc} -b & (\lambda -p_{c}eb)/2\\ (\lambda -p_{c}eb)/2 & p_{c}e\lambda -k \end{array}\right]$. Because $D_{1}=-b<0$, when $D_{2}=[4bk-{\left(\lambda +p_{c}eb\right)}^{2}]/4>0$, $H_{2}(w,\beta )$ is a negative definite matrix, and $E(\pi_{m}^{n})$ is a concave function of w and β. Setting $\partial E(\pi_{m}^{n})/\partial w=0$, $\partial E(\pi_{m}^{n})/\partial \beta =0$, and parallel to the equations, the optimal $w^{n}$ and $\beta^{n}$ under the risk-neutral decentralized decision of the manufacturer can be obtained: $w^{n}=\frac{2k(\bar{s}+bc+p_{c}eb)-(\lambda +p_{c}eb)(p_{c}e\bar{s}+p_{c}e\lambda +c\lambda )}{4bk-{\left(\lambda +p_{c}eb\right)}^{2}}$, $\beta^{n}=\frac{\left(\bar{s}-bc-p_{c}eb)(\lambda +p_{c}eb\right)}{4bk-{\left(\lambda +p_{c}eb\right)}^{2}}$. Substituting $w^{n}$ and $\beta^{n}$ into the expressions of q and $p^{n}$, the optimal order quantity and selling price of the manufacturer under the risk-neutral decentralized decision can be obtained as follows: $q^{n}=\frac{bk(\bar{s}-bc-p_{c}eb)}{4bk-{\left(\lambda +p_{c}eb\right)}^{2}}$, $p^{n}=\frac{k(3\bar{s}+bc+p_{c}eb)-(\lambda +p_{c}eb)(p_{c}e\bar{s}+p_{c}e\lambda +c\lambda )}{4bk-{\left(\lambda +p_{c}eb\right)}^{2}}$. □

## Appendix C

**Proof** **of** **Proposition** **3.** Under the manufacturer’s risk aversion, the retailer and manufacturer also follow the Stackelberg game process. We use the reverse derivation method, and the retailer as the follower determines the optimal unit product selling price p according to the manufacturer’s decision. Since $\partial^{2}U(\pi_{r}^{m})/\partial p^{2}=-2b<0$, let $\partial U(\pi_{r}^{m})/\partial p=0$; then, the reaction function of p on w and β can be obtained: $p^{m}=(\bar{s}+\lambda \beta +bw)/2b$. Substituting $p^{m}$ into $U(\pi_{m}^{m})$, the Hessian matrix $H_{2}(w,\beta )$ of $U(\pi_{m}^{m})$ regarding w and β can be expressed as $H_{3}(w,\beta )=\left[\begin{array}{cc} -b & (\lambda -p_{c}eb)/2\\ (\lambda -p_{c}eb)/2 & p_{c}e\lambda -k \end{array}\right]$. Because $D_{1}=-b<0$, when $D_{2}=[4bk-{\left(\lambda +p_{c}eb\right)}^{2}]/4>0$, $H_{3}(w,\beta )$ is a negative definite matrix, and $U(\pi_{m}^{m})$ is a concave function of w and β. Setting $\partial U(\pi_{m}^{m})/\partial w=0$, $\partial U(\pi_{m}^{m})/\partial \beta =0$, and parallel to the equations, w and β under the decentralized decision of manufacturer risk aversion can be obtained: $w^{m}=\frac{2k(\bar{s}+bc+p_{c}eb-2\eta \delta_{s})-(\lambda +p_{c}eb)(p_{c}e\bar{s}+p_{c}e\lambda +c\lambda -2\eta \delta_{s}p_{c}e)}{4bk-{\left(\lambda +p_{c}eb\right)}^{2}}$, $\beta^{m}=\frac{\left(\bar{s}-bc-p_{c}eb-2\eta \delta_{s})(\lambda +p_{c}eb\right)}{4bk-{\left(\lambda +p_{c}eb\right)}^{2}}$. Substituting $w^{m}$ and $\beta^{m}$ into the expressions of q and $p^{m}$, the optimal order quantity and selling price under the decentralized decision of the manufacturer with risk aversion can be obtained as follows: $q^{m}=\frac{bk(\bar{s}-bc-p_{c}eb)+\eta \delta_{s}[2bk-{\left(\lambda +p_{c}eb\right)}^{2}]}{4bk-{\left(\lambda +p_{c}eb\right)}^{2}}$, $p^{m}=\frac{bk(3\bar{s}+bc+p_{c}eb-2\eta \delta_{s})-(\lambda +p_{c}eb)(p_{c}eb\bar{s}+p_{c}eb\lambda +cb\lambda -p_{c}eb\eta \delta_{s}+\eta \delta_{s}\lambda )}{b[4bk-{\left(\lambda +p_{c}eb\right)}^{2}]}$. Obviously, when $2k(\bar{s}+bc+p_{c}eb-2\eta \delta_{s})-(\lambda +p_{c}eb)(p_{c}e\bar{s}+p_{c}e\lambda +c\lambda -2\eta \delta_{s}p_{c}e)>0$ and $\bar{s}-bc-p_{c}eb>2\eta \delta_{s}$, $w^{m}$, $\beta^{m}$, $q^{m}$, and $p^{m}$ are nonnegative. Solve the inequality at this time and set $\eta <\frac{\left[2k(\bar{s}+bc+p_{c}eb)-(\lambda +p_{c}eb)(p_{c}e\bar{s}+p_{c}e\lambda +c\lambda )\right]}{2\delta_{s}[2k-(\lambda +p_{c}eb)p_{c}e]}=\eta_{1}$ and $\eta <\frac{\bar{s}-bc-p_{c}eb}{2\delta_{s}}=\eta_{2}$. Since $\eta_{1}-\eta_{2}=\frac{\left(p_{c}e+c)[4bk-{\left(\lambda +p_{c}eb\right)}^{2}\right]}{2\delta_{s}[2k-(\lambda +p_{c}eb)p_{c}e]}>0$, then $\eta <\frac{\bar{s}-bc-p_{c}eb}{2\delta_{s}}$. □

## Appendix D

**Proof** **of** **Proposition** **7.** Since $\partial^{2}E(\pi_{rn}^{c})/\partial p^{2}=-2b<0$, let $\partial E(\pi_{rn}^{c})/\partial p=0$; then, the reaction function of p on w and β can be obtained: $p_{n}^{c}=(\bar{s}+\lambda \beta +bw)/2b$. Substitute $p_{n}^{c}$ into $E(\pi_{mn}^{c})$, $E(\pi_{mn}^{c})$. The Hessian matrix $H_{4}(w,\beta )$ of w and β can be expressed as: $H_{4}(w,\beta )=\left[\begin{array}{cc} -b & (\lambda -p_{c}eb)/2\\ (\lambda -p_{c}eb)/2 & p_{c}e\lambda -k(1-\phi ) \end{array}\right]$. Because $D_{1}=-b<0$, when $D_{2}=[4bk(1-\phi )-{\left(\lambda +p_{c}eb\right)}^{2}]/4>0$, $H_{4}(w,\beta )$ is a negative definite matrix, and $E(\pi_{mn}^{c})$ is a concave function of w and β. Set $\partial E(\pi_{mn}^{c})/\partial w=0$, $\partial E(\pi_{mn}^{c})/\partial \beta =0$, and parallel to the equations, w and β in the manufacturer’s risk-neutral contract coordination decision can be obtained: $w_{n}^{c}=\frac{2k(1-\phi )(\bar{s}+bc+p_{c}eb)-(\lambda +p_{c}eb)(p_{c}es+p_{c}e\lambda +c\lambda )}{4bk(1-\phi )-{\left(\lambda +p_{c}eb\right)}^{2}}$, $\beta_{n}^{c}=\frac{\left(\bar{s}-bc-p_{c}eb)(\lambda +p_{c}eb\right)}{4bk(1-\phi )-{\left(\lambda +p_{c}eb\right)}^{2}}$. Substituting $w_{n}^{c}$ and $\beta_{n}^{c}$ into the expressions of q and $p_{n}^{c}$, the q and p for the manufacturer’s risk-neutral contract coordination decision can be obtained as follows: $p_{n}^{c}=\frac{k(1-\phi )(3\bar{s}+bc+p_{c}eb)-(\lambda +p_{c}eb)(p_{c}e\bar{s}+p_{c}e\lambda +c\lambda )}{4bk(1-\phi )-{\left(\lambda +p_{c}eb\right)}^{2}}$, $q_{n}^{c}=\frac{bk(1-\phi )(\bar{s}-bc-p_{c}eb)}{4bk(1-\phi )-{\left(\lambda +p_{c}eb\right)}^{2}}$. □

## Appendix E

**Proof** **of** **Proposition** **8.** Since $\partial^{2}U(\pi_{rm}^{c})/\partial p^{2}=-2b<0$, let $\partial U(\pi_{rm}^{c})/\partial p=0$, and the response function of p on w and β can be obtained: $p_{m}^{c}=(\bar{s}+\lambda \beta +bw)/2b$. Substituting $p_{m}^{c}$ into $U(\pi_{mm}^{c})$, the Hessian matrix of $H_{5}(w,\beta )$ of w and β of $U(\pi_{mm}^{c})$ can be expressed as $H_{5}(w,\beta )=\left[\begin{array}{cc} -b & (\lambda -p_{c}eb)/2\\ (\lambda -p_{c}eb)/2 & p_{c}e\lambda -k(1-\phi ) \end{array}\right]$. Because $D_{1}=-b<0$, when $D_{2}=[4bk(1-\phi )-{\left(\lambda +p_{c}eb\right)}^{2}]/4>0$, $H_{5}(w,\beta )$ is a negative definite matrix, and $U(\pi_{mm}^{c})$ is the concave function of w and β. Let $\partial U(\pi_{mm}^{c})/\partial w=0$, $\partial U(\pi_{mm}^{c})/\partial \beta =0$ and set up equations in parallel to obtain the optimal wholesale price and carbon emission reduction rate of the manufacturer’s risk aversion contract coordination decision: $w_{m}^{c}=\frac{2k(1-\phi )(\bar{s}+bc+p_{c}eb-2\eta \delta_{s})-(\lambda +p_{c}eb)(p_{c}e\bar{s}+p_{c}e\lambda +c\lambda -2\eta \delta_{s}p_{c}e)}{4bk(1-\phi )-{\left(\lambda +p_{c}eb\right)}^{2}}$, $\beta_{m}^{c}=\frac{\left(\bar{s}-bc-p_{c}eb-2\eta \delta_{s})(\lambda +p_{c}eb\right)}{4bk(1-\phi )-{\left(\lambda +p_{c}eb\right)}^{2}}$. Substituting $w_{m}^{c}$ and $\beta_{m}^{c}$ into q and $p_{m}^{c}$, the optimal order quantity and selling price for the manufacturer’s risk avoidance contract coordination decision can be obtained as follows: $q_{m}^{c}=\frac{bk(1-\phi )(\bar{s}-bc-p_{c}eb)+\eta \delta_{s}[2bk(1-\phi )-{\left(\lambda +p_{c}eb\right)}^{2}]}{4bk(1-\phi )-{\left(\lambda +p_{c}eb\right)}^{2}}$, $p_{m}^{c}=\frac{bk(1-\phi )(3\bar{s}+bc+p_{c}eb-2\eta \delta_{s})-(\lambda +p_{c}eb)(p_{c}eb\bar{s}+p_{c}eb\lambda +cb\lambda -p_{c}eb\eta \delta_{s}+\eta \delta_{s}\lambda )}{b[4bk(1-\phi )-{\left(\lambda +p_{c}eb\right)}^{2}]}$. □

## Appendix F

**Proof** **of** **Proposition** **9.** (i) Carbon emission reduction cost-sharing contract to achieve Pareto improvement, manufacturers need to meet $\pi_{mn}^{c}>\pi_{m}^{n}$, retailers need to meet $\pi_{rn}^{c}>\pi_{r}^{n}$. From $\pi_{mn}^{c}>\pi_{m}^{n}$ we can derive $\phi <\frac{4bk-{\left(\lambda +p_{c}eb\right)}^{2}}{4bk}=\phi_{1}$; From $\pi_{rn}^{c}>\pi_{r}^{n}$ we can derive $\phi <\frac{{\left(\lambda +p_{c}eb\right)}^{2}[4bk-{\left(\lambda +p_{c}eb\right)}^{2}]}{2bk[8bk-{\left(\lambda +p_{c}eb\right)}^{2}]}=\phi_{2}$. Because $2bk-{\left(\lambda +p_{c}eb\right)}^{2}>0$, we can easily get $\phi_{1}-\phi_{2}>0$. Combined with the feasibility condition of ϕ: 0<ϕ<1, the cost-sharing coefficient range of the manufacturer’s risk-neutral decision can be obtained: $0<\phi <\frac{{\left(\lambda +p_{c}eb\right)}^{2}[4bk-{\left(\lambda +p_{c}eb\right)}^{2}]}{2bk[8bk-{\left(\lambda +p_{c}eb\right)}^{2}]}$. (ii) The second derivative of the cost-sharing coefficient ϕ. with respect to $\pi_{rn}^{c}$ can be obtained: $\frac{\partial^{2}\pi_{rn}^{c}}{\partial \phi^{2}}=\frac{-2bk^{2}{\left(\bar{s}-bc-p_{c}eb\right)}^{2}{\left(\lambda +p_{c}eb\right)}^{2}[8bk+16bk\phi -5{\left(\lambda +p_{c}eb\right)}^{2}]}{{\left[4bk(1-\phi )-{\left(\lambda +p_{c}eb\right)}^{2}\right]}^{4}}$. Obviously, when the cost-sharing coefficient $\phi >\frac{5{\left(\lambda +p_{c}eb\right)}^{2}-8bk}{16bk}$, $\frac{\partial^{2}\pi_{rn}^{c}}{\partial \phi^{2}}<0$. And $\frac{{\left(\lambda +p_{c}eb\right)}^{2}[4bk-{\left(\lambda +p_{c}eb\right)}^{2}]}{2bk[8bk-{\left(\lambda +p_{c}eb\right)}^{2}]}=M_{1}$, $\frac{{5(\lambda +p_{c}eb)}^{2}-8bk}{16bk}=M_{2}$, $M_{1}-M_{2}=\frac{\left[8bk-3{\left(\lambda +p_{c}eb\right)}^{2}][8bk+{\left(\lambda +p_{c}eb\right)}^{2}\right]}{16bk[8bk-{\left(\lambda +p_{c}eb\right)}^{2}]}$. Because $2bk-{\left(\lambda +p_{c}eb\right)}^{2}>0$, you can easily get $M_{1}-M_{2}>0$. Therefore, when $\frac{5{\left(\lambda +p_{c}eb\right)}^{2}-8bk}{16bk}<\phi <\frac{{\left(\lambda +p_{c}eb\right)}^{2}[4bk-{\left(\lambda +p_{c}eb\right)}^{2}]}{2bk[8bk-{\left(\lambda +p_{c}eb\right)}^{2}]}$, $\frac{\partial^{2}\pi_{rn}^{c}}{\partial \phi^{2}}<0$. At this point, let $\frac{\partial \pi_{rn}^{c}}{\partial \phi }=0$, the optimal cost-sharing coefficient determined by the retailer can be $\phi^{*}=\frac{{\left(\lambda +p_{c}eb\right)}^{2}}{8bk}$. (iii) When $0<\phi \leq \frac{5{\left(\lambda +p_{c}eb\right)}^{2}-8bk}{16bk}$, $\frac{\partial^{2}\pi_{rn}^{c}}{\partial \phi^{2}}\geq 0$. At this point, the retailer’s profit function $\pi_{rn}^{c}$ is the lower convex function of the cost-sharing coefficient ϕ, so the smaller $\phi^{*}$ is, the better. □

## Appendix G

**Proof** **of** **Proposition** **10.** Let bk=B, ${\left(\lambda +p_{c}eb\right)}^{2}=C$, $(\bar{s}-bc-p_{c}eb)=S$, $\eta \delta_{s}=H$, (i) The carbon emission reduction cost-sharing contract realizes Pareto improvement. Manufacturers need to meet $\pi_{mm}^{c}>\pi_{m}^{m}$, and retailers need to meet $\pi_{rm}^{c}>\pi_{r}^{m}$. From $\pi_{mm}^{c}>\pi_{m}^{m}$, $\phi <\frac{4B-C}{4B}=\phi_{3}$ can be derived, and from $\pi_{rm}^{c}>\pi_{r}^{m}$, $\phi <\frac{\left(4B-C)[CS+(16B-6C)H\right]}{2B[(8B-C)S+(16B-6C)H]}=\phi_{4}$ can be derived. Comparing the cost-sharing coefficients $\phi_{3}$ and $\phi_{4}$, there is $\phi_{3}-\phi_{4}=\frac{\left(4B-C)(S-2H)(8B-3C\right)}{4B[(8B-C)S+(16B-6C)H]}$. From the previous feasibility conditions 2B−C>0, S>2H, it is easy to get $\phi_{3}-\phi_{4}>0$. Combining the feasibility condition of ϕ: 0<ϕ<1, the range of the cost-sharing coefficient of the manufacturer’s risk-aversion decision can be obtained: $0<\phi <\frac{\left(4B-C)[CS+(16B-6C)H\right]}{2B[(8B-C)S+(16B-6C)H]}$. (ii) Find the second derivative of the cost-sharing coefficient ϕ for $\pi_{rm}^{c}$ to obtain $\frac{\partial^{2}\pi_{rm}^{c}}{\partial \phi^{2}}=\frac{-2B^{2}C(S-2H)[8BS+16BS\phi -5CS-3H(16B-6C)]}{b{\left[4B(1-\phi )-C\right]}^{4}}$. Obviously, when the cost-sharing coefficient $\phi >\frac{5CS+3H(16B-6C)-8BS}{16BS}$, $\frac{\partial \pi_{rm}^{c}}{\partial \phi^{2}}<0$. Let $\frac{\left(4B-C)[CS+(16B-6C)H\right]}{2B[(8B-C)S+(16B-6C)H]}=N_{1}$, $\frac{5CS+3H(16B-6C)-8BS}{16BS}=N_{2}$, $N_{1}-N_{2}=\frac{\left[(8B-3C)(S-2H)][(8B+C)S+3H(16B-6C)\right]}{16BS[(16B-6C)H+(8B-C)S]}$. Because 2B−C>0 and S>2H, it is easy to get $N_{1}-N_{2}>0$. Therefore, when $\frac{5CS+3H(16B-6C)-8BS}{16BS}<\phi <\frac{\left(4B-C)[CS+(16B-6C)H\right]}{2B[(8B-C)S+(16B-6C)H]}$, $\frac{\partial^{2}\pi_{rm}^{c}}{\partial \phi^{2}}<0$. At this time, let $\frac{\partial \pi_{rm}^{c}}{\partial \phi }=0$, the optimal cost-sharing coefficient determined by the retailer can be obtained as $\phi^{*}=\frac{CS+(16B-6C)H}{8BS}$. (iii) When $0<\phi \leq \frac{5CS+3H(16B-6C)-8BS}{16BS}$, $\frac{\partial^{2}\pi_{rm}^{c}}{\partial \phi^{2}}\geq 0$. At this time, the retailer’s profit function $\pi_{rm}^{c}$ is a downward convex function of the cost-sharing coefficient ϕ, so the smaller $\phi^{*}$ is, the better. □
